# Clinical and Economic Evaluation of Salvianolate Injection for Coronary Heart Disease: A Retrospective Study Based on National Health Insurance Data in China

**DOI:** 10.3389/fphar.2020.00887

**Published:** 2020-06-18

**Authors:** Lisong Yang, Xiaolan Chen, Carolina Oi Lam Ung, He Zhu, Hao Hu, Sheng Han

**Affiliations:** ^1^State Key Laboratory of Quality Research in Chinese Medicine, Institute of Chinese Medical Sciences, University of Macau, Macau, Macau; ^2^International Research Center for Medicinal Administration, Peking University, Beijing, China; ^3^School of Physics and Telecommunication Engineering, South China Normal University, Guangzhou, China

**Keywords:** salviaolate injection, coronary heart disease, clinical evaluation, economic evaluation, Danhong injection, alprostadil injection

## Abstract

**Objective:**

The study aimed to conduct clinical and economic evaluation of salvianolate injection for patients with coronary heart disease (CHD) in comparison to Danhong injection and alprostadil injection.

**Method:**

This was a retrospective study using National Health Insurance Data about inpatients diagnosed with CHD in China in 2015 who met the inclusion criteria. The recruited patients were divided into two samples: surgery and non-surgery. The exposed group received salvianolate injection, while the control group received either alprostadil injection or Danhong injection. The medical cost per hospitalization, hospitalization duration, and the rehospitalization rates were used as outcome indicators. Heterogeneity was processed according to disease stratification. Propensity score matching and multivariate analysis were used for statistical analysis to control potential confounding factors.

**Results:**

The hospitalization duration of salvianolate injection group was significantly (*P* < 0.05) shorter than that of Danhong injection group in the non-surgery sample. The hospitalization duration of salvianolate injection group was significantly (*P* < 0.05) shorter than those of alprostadil injection group in both surgery and non-surgery samples. In the non-surgery sample, the medical cost per hospitalization of salvianolate injection group was significantly (*P* < 0.05) lower than that of alprostadil injection group. However, there were no statistical differences of rehospitalization rates in salvianolate injection group versus alprostadil injection group or salvianolate injection group versus Danhong injection group in both surgery and non-surgery samples.

**Conclusion:**

Salvianolate injection showed advantages in reducing hospitalization duration for inpatients with CHD when comparing with alprostadil injection and Danhong injection. The results of this real-world study can help to inform clinical practice for CHD patients.

## Introduction

Coronary atherosclerotic heart disease is a heart disease caused by myocardial ischemia, hypoxia, or necrosis. Its underlying cause is that coronary atherosclerosis causes stenosis, spasm, or obstruction of the coronary lumen. They are collectively referred as coronary heart disease (CHD) or coronary artery disease, called CHD for short (Chen, 2013). In recent years, the morbidity and prevalence rate of coronary disease in China has been increasing significantly, especially the young people’s death of coronary disease gets more and more attention (Wan et al., 2012; Xu et al., 2018). According to the research data of the Lancet in 2013, the death rate of CHD in China increased by 31.6% from 1990 to 2010, and CHD rose from the seventh place to the second place in the list of causes of premature death in China (Yang et al., 2013). CHD can affect the quality of life of patients severely, for example, the incidence of sudden death in people with a record of CHD attack is 4 or 6 times higher than that in the general population (Klootwijk and Hamm, 1999). With the trend of patients being young, the possibility of disability in patients with CHD is also significantly increased. At the same time, all kinds of medical costs of CHD are enormous because of a long course, a low cure rate, and a high recurrence rate. In China, the average cost of interventional therapy for CHD is about 40,000 to 60,000 CNY per time, and the cost of coronary artery bypass grafting is about 40,000 to 100,000 CNY (Liu, 2008).

Salvia miltiorrhiza and its compound preparations have the effect of improving blood flow, activating blood circulation to dissipate blood stasis (Zhou et al., 2005; Wang and Xuan, 2008). Known as Danshen, they have been used in China for a long history with a wide variety and a large amount of consumption. They are extensively used in the treatment of cardiovascular diseases, such as CHD, angina pectoris, hyperlipidemia, and acute ischemic stroke (Walden and Tomlinson, 2011). In Japan, the United States, and some European countries, products of Salvia miltiorrhiza also can be obtained.

Traditional Chinese medicine injections (TCMIs) are produced from herbals using modern techniques and have been widely used in the clinic in China (China PC, 2015). Salvianolate injection is one of the most extensive prescription drugs used in the treatment of cardiovascular disease since it was launched in 2005 in China. Some TCMIs may contain multiple unknown compounds, but the components of salvianolate injection were clarified, which may lead to less severe anaphylaxis. Some clinical trials and systematic evaluations have confirmed that salvianolate injection can substantially improve the clinical symptoms of patients with CHD (Sun, 2014; Liu et al., 2016; Li, 2016), and can reduce the angina attacks with functional safety, low incidence of adverse drug reactions (Li and Huang, 2016; Feng et al., 2016; He et al., 2016; Zhang et al., 2016), which shows that salvianolate injection is well tolerated in the general population (Yan et al., 2017).

However, there are few clinical and economic evaluations of salvianolate injection in real-world practice. Some retrospective studies had compared salvianolate injection with other salvia miltiorrhiza injections (Li and Yin, 2010; Yu and Yan, 2012; Shi et al., 2014). Nevertheless, in clinical practice, doctors tend to choose drugs on consideration of patients’ actual conditions. Therefore, to evaluate the clinical and economic effects of specific Chinese medicine products must consider realistic comparator drugs (Tian and Xie, 2010). In current literature, the evaluation of curative effect and economic benefit of the salvianolate injection with the comparison of other Chinese medicine products and chemical drugs for CHD patients in the real clinical environment is still lacking (Dong et al., 2018).

In current clinic practice, Danhong injection is a useful clinical medication for CHD and angina. It has a good curative effect with little adverse reaction (Zheng and Luo, 2013; Sun and Xie, 2014; Li et al., 2018). Besides, alprostadil injection can significantly improve myocardial microcirculatory disturbance and hemorheological disorder in patients with CHD, which is also used for CHD in the current clinic (Huang et al., 2017). However, in the literature, there are few comparative economic and clinical evaluation of Chinese patent medicine and chemical medicine in the treatment of CHD.

Therefore, this study aimed to conduct clinical and economic evaluation of salvianolate injection for patients with CHD in comparison to Danhong injection and alprostadil injection. It is expected that the findings can provide evidence for clinical treatment for CHD.

## Methods

### Composition Information

On 25^th^ May 2015, salvianolate injection was approved by the State Food and Drug Administration (SFDA) of China as a new drug (Batch Number: Z20050247-49). First authorized on 4^th^ June 2007 to the Shanghai Green Valley Pharmaceutical Co, Ltd to manufacture, salvianolate injection was renewed on 15^th^ July 2016 by SFDA.

#### Botanical

The species was named as Salvia miltiorrhiza Bunge (Family: Lamiaceae Martinov, Genus: Salvia L.), which is accepted by the Kew Medicinal plant names service, and its native range is Central & S. China to Vietnam (Kew, 2020). First published in Enum. Pl. China Bor.: 50 (1833), Salvia miltiorrhiza Bunge (Bge.) was cited by a lot of medicinal references such as Pharmacopoeia of China (2015), British Pharmacopoeia 2015, Vol. 4 (2014), U.S. Pharmacopoeia USP 39 (2016), and U.S. FDA Substance Registration System (2016) (British Pharmacopoeia Commission, 2015; China PC, 2015; United States Pharmacopeial Convention, 2016; U.S. FDA, 2016).

#### Chemical

Salvianolate injection is a traditional Chinese medicine injection identified by the SFDA, containing major homologues such as salvianolic acid B (≥85%), rosemary acid (≥10.1%), and lithospermic acid (≥1.9%).

Since the early 1930s, China has begun to study the chemical composition of salvia miltiorrhiza. However, these early studies focused on lipophilic compounds (Zhou et al., 2005). With the support of the Chinese Academy of Sciences and the Ministry of Science and Technology of PRC, the Shanghai Institute of Materia Medica found that the water-soluble active ingredients of salvia miltiorrhiza existed in the form of salts. Through active screening and pharmacological studies, it was found that active compounds are mainly salvianolic acid B and homologs, such as rosemary acid, lithospermic acid, dansensu, salvianolic acid B, tanshinone IIA, and dimethyl lithospermate (Chan et al., 2004; Kang et al., 2004; Li et al., 2009), among which salvianolic acid B had the strongest pharmacological effect. It was also clarified that salvianolic acid B was the most important active ingredients of salvia miltiorrhiza in the treatment of cardiovascular diseases. A method for extracting and purifying chemical constituents from salvia miltiorrhiza by ethanol extraction method was well established, related technologies have been approved as Chinese patents and United States patents.

### Target Population and Data Source

The target population of this study is CHD patients who were followed up from the first inpatient treatment in 2015 to December 31, 2015. The data was collected from the National Urban Basic Medical Insurance Database at the China Medical Insurance Research Association. China Medical Insurance Research Association is a national, semi-official organization that is directly managed by the National Healthcare Security Administration of China. With the official support of the National Healthcare Security Administration of China, China Medical Insurance Research Association establishes and maintains the National Urban Basic Medical Insurance Database that regularly and systematically collects the reimbursement data of social insurance from all the provincial governments in China (China Medical Insurance Research Association). Therefore, the National Urban Basic Medical Insurance Database at the China Medical Insurance Research Association is one of the most reliable databases for retrospective study in China.

The specific inclusion criteria of the patients are:

1. Patient who diagnosed with CHD for the first time before September 30, 2015;
2. CHD was the principal diagnosis (identification terms: angina pectoris, CHD, coronary artery, atherosclerosis, cardiac or myocardial infarction, ICD-10 code: I20-I25) when discharged from inpatient department and frequency of visits occurred before September 30, 2015.

The specific exclusion criteria of the patients are:

1. Patients who younger than or equal to 18 years old;
2. Patients with malignant tumors or schizophrenic patients or patients on dialysis.

### Treatment and Grouping

All patients were divided into exposed group and control group. The exposed group was hospitalized with salvianolate injection, and the control group was treated with Danhong injection or alprostadil injection. The outcomes of salvianolate injection and Danhong injection, salvianolate injection, and alprostadil injection were compared.

- Salvianolate Injection Group
  The principal diagnosis during hospitalization was CHD and was prescribed salvianolate injection while alprostadil injection and other traditional Chinese medicine injections of blood-activating and stasis-dissolving are not prescribed.
- Danhong Injection Group
  The principal diagnosis during hospitalization was CHD and was prescribed Danhong injection while alprostadil injection and other traditional Chinese medicine injections of blood-activating and stasis-dissolving are not prescribed.
- Alprostadil Injection Group
  The principal diagnosis during hospitalization was CHD and was prescribed alprostadil injection while other traditional Chinese medicine injections of blood-activating and stasis-dissolving are not prescribed.

### Study Perspective and Cost

This study chose a perspective of medical insurance payer. All the costs were only based on the reimbursement records of medical insurance. Compared with the medical record data at hospitals, the insurance claim data in China has few risk and cost parameters. Therefore, this study focused on the medical costs per hospitalization because this data is most reliable data to reflect the economic effectiveness of treatments.

### Outcomes

The outcome indicators of this study include medical cost per hospitalization, hospitalization duration, 10d re-hospitalization rate, 30d re-hospitalization rate, and 90d re-hospitalization rate. Their definitions are listed below:

- 10d Rehospitalization Rate: refers to the proportion of inpatients with CHD who re-hospitalized within 10 days of the total number of hospitalized patients in this group after the first hospitalization. (If there are more than one readmission for CHD, counted as rehospitalization)
- 30d Rehospitalization Rate: refers to the proportion of inpatients with CHD who re-hospitalized within 30 days of the total number of hospitalized patients in this group after the first hospitalization. (If there are more than one readmission for CHD, counted as rehospitalization)
- 90d Rehospitalization Rate: refers to the proportion of inpatients with CHD who re-hospitalized within 90 days of the total number of hospitalized patients in this group after the first hospitalization. (If there are more than one readmission for CHD, counted as rehospitalization)

### Bias and Elimination

The primary bias of this study was the selection bias. Thus, the Propensity Score Matching (PSM) method was used to control the confounding factors and reduce the impact of confounding factors on the intervention effect estimation.

In this study, the surgery sample was covariate based on age, gender, region, level of medical institution, whether combined with stroke, whether nitrate esters are prescribed (ATC code: XC01DA), whether nitrate esters are prescribed (ATC code: XB01AD), whether percutaneous coronary intervention (PCI) or coronary artery bypass grafting (CABG) was used. The matching ratio is 1:1, and the caliper value is 0.05. Furthermore, the non-surgery sample was covariate based on age, gender, region, level of medical institution, whether combined with stroke, whether nitrate esters are prescribed (ATC code: XC01DA), whether nitrate esters are prescribed (ATC code: XB01AD). The matching ratio is 1:1, and the caliper value is 0.05.

### Heterogeneity

In order to avoid or reduce the impact of disease severity on the results, stratified analysis was adopted in this study to divide the population of CHD patients into surgery sample and non-surgery sample according to whether they had received surgery during hospitalization, and the difference of outcome indicators between the exposure group and the two control groups was analyzed in each sample.

### Statistical Analysis

Descriptive analysis: measurement data are described by mean ± standard deviation (x ± s) or median and quartile, and enumeration data are described by frequency and rate.

Single factor analysis: For measurement data, the indicators of exposure group and control group were compared, T-test was used to compare the indicators between the exposed group and the control group which obeyed the normal distribution, Wilcoxon rank sum test was used to compare the two groups of indicators that did not obey the normal distribution. For enumeration data, Chi-square test was used. For all the above, *P* < 0.05 was considered statistically significant.

Multiple-factor analysis: Multivariate linear regression model was used for the dependent variable of hospitalization cost per hospitalization and hospitalization duration. If the dependent variable did not obey normal distribution, then run a natural logarithmic transformation. If the variance was not homogeneous, then do robust regression adjustment.

## Results

**Figure 1:**
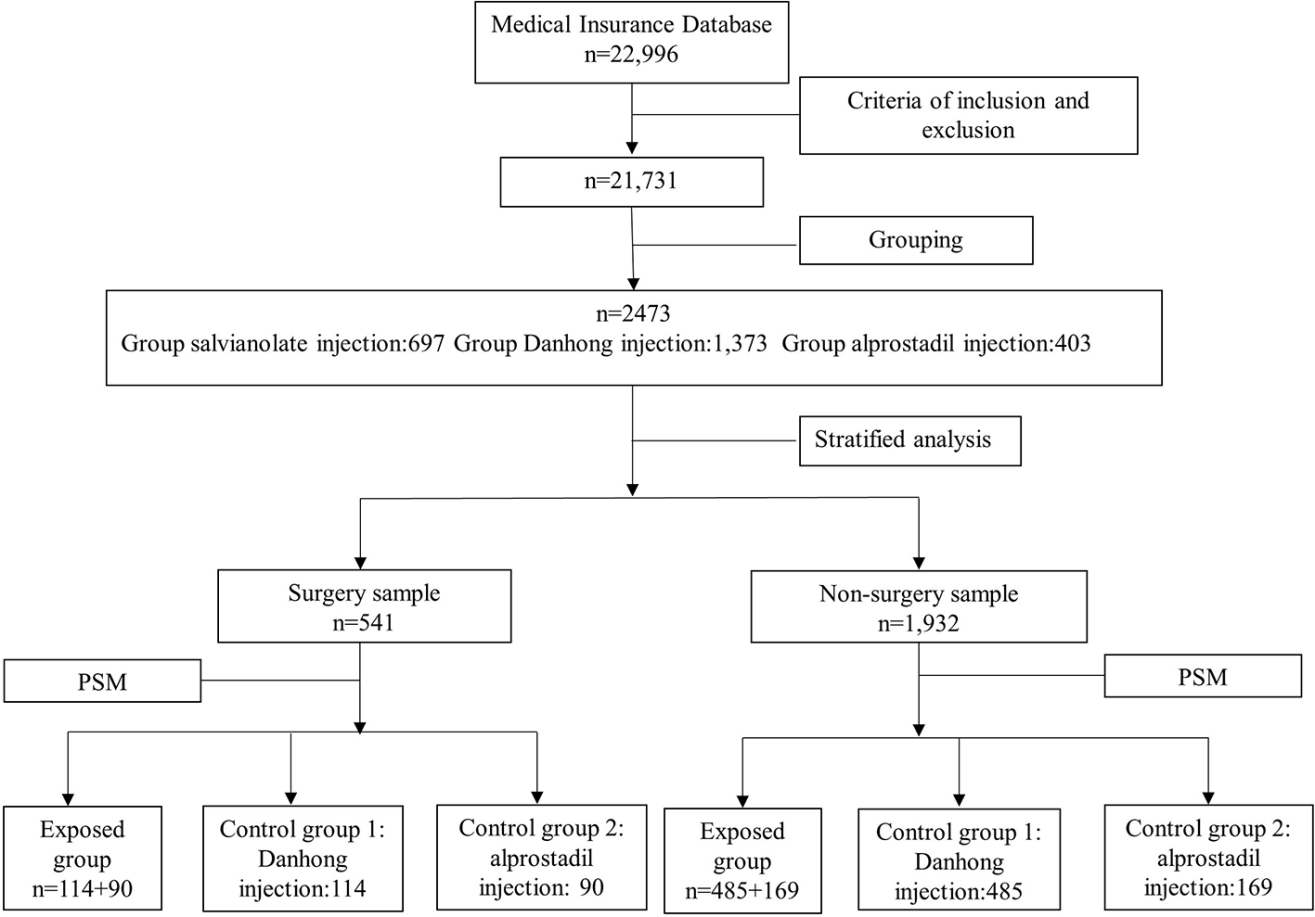
Flowchart of sampling and grouping. The unit in the figure is per person.

In this study, 2,473 patients with CHD who met the inclusion and exclusion criteria were included, including 541 patients in the surgery sample and 1,932 patients in the non-surgery sample. The flowchart of sampling and grouping are summarized in **Figure 1**.

### Baseline Characteristics of the Total Sample

The difference between the surgery sample and the non-surgery sample is statistically significant in the following variables: age, gender, level of medical institutions, regional distribution, whether comorbid with high blood pressure or heart failure or diabetes, whether combine medication with beta blockers or nitrate esters or angiotensin converting enzyme inhibitors (ACEI) or angiotensin receptor blockers (ARB) or antiplatelet/thrombolytic/anticoagulant or lipid-regulating drugs.

Among them, the age of the non-surgery sample is slightly higher than that of the surgery sample. There are more males in the surgery sample than in the non-surgery sample. The surgery sample mainly distributes in tertiary hospitals and eastern region, while the non-surgery sample mainly distributes in secondary and grass-roots hospitals and central and western region. The proportion of patients with high blood pressure, heart failure, and diabetes in the surgery sample was higher than that in the non-surgery sample, and the proportion of patients with drugs combined in the surgery sample was higher than that in the non-surgery sample except for calcium channel blockers. Among the hospitalized patients with CHD, 170 patients had undergone CHD related surgery (PCI/CABG), accounting for 6.9% (as shown in **Table 1**).

**Table 1:** Overall baseline characteristics of hospitalized patients with CHD.

Variable	Total		Surgery sample		Non-surgery sample		*Z/χ^2^*	*P*
	(N = 2473)		(N = 541)		(N = 1932)			
	N	%	N	%	N	%		
Age, P_50_ (P_25_-P_75_)	68 (60–76)		67 (60–74)		68 (60–77)		2.39	0.0168
Gender							56.14	<0.001
Male	1225	49.5	345	63.8	880	45.6		
Female	1248	50.5	196	36.2	1052	54.5		
Level of medical institutions							206.46	<0.001
Tertiary hospital	1006	40.7	359	66.4	647	33.5		
Secondary hospital	1022	41.3	157	29.0	865	44.8		
Primary hospital	445	18.0	25	4.6	420	21.7		
Region							52.21	<0.001
Eastern region	1046	42.3	301	55.6	745	38.6		
Central region	1187	48.0	192	35.5	995	51.5		
Western region	240	9.7	48	8.9	192	9.9		
Comorbidity								
High blood pressure	700	28.3	205	37.9	495	25.6	31.36	<0.001
Heart failure	163	6.6	50	9.2	113	5.9	7.90	0.0050
Atrial fibrillation	50	2.0	7	1.3	43	2.2	1.85	0.1735
Heart valve disease	9	0.4	4	0.7	5	0.3	2.69	0.1009
Stroke	181	7.3	43	8.0	138	7.1	0.40	0.5250
Pulmonary embolism	6	0.2	0	0.0	6	0.3	1.68	0.1944
COPD	38	1.5	8	1.5	30	1.6	0.02	0.9015
chronic nephrosis	27	1.1	8	1.5	19	1.0	0.96	0.3271
Diabetes	643	26.0	215	39.7	428	22.2	67.95	0.0001
Drug combination								
Beta blockers	1182	47.8	342	63.2	840	43.5	65.99	0.0001
Nitrate esters	1932	78.1	506	93.5	1426	73.8	96.18	0.0001
ACEI or ARB	1141	46.1	307	56.8	834	43.2	31.36	0.0001
Calcium antagonist	922	37.3	199	36.8	723	37.4	0.07	0.7860
Antiplatelet/thrombolytic/anticoagulant	1428	57.7	476	88.0	952	49.3	259.55	0.0001
Lipid regulating agents	1817	73.5	473	87.4	1344	69.6	69.21	0.0001
Related surgery of CHD								
PCI/CABG	170	6.9	170	31.4				

### Surgery Sample: Salvianolate Injection and Danhong Injection

#### Baseline Characterization

After stratified analysis and PSM, 114 pairs of matched patients were obtained. In the surgery sample, the salvianolate injection group and the Danhong injection group were all 114 patients. There were statistically significant differences in the distribution of beta blockers and ACEI or ARB drugs between the two groups. The differences between the two groups were not statistically significant in the following variables: age, gender, level of medical institutions, regional distribution, whether comorbid with other diseases, whether combine medication with other drugs and whether had undergone CHD related surgery (as shown in **Table 2**).

**Table 2:** Baseline characteristics of salvianolate injection group and Danhong injection group in the surgery sample.

Variable	Total		Salvianolate injection group		Danhong injection group		*Z/χ^2^*	*P*
	(N = 228)		(N = 114)		(N = 114)			
	N	%	N	%	N	%		
Age, P_50_ (P_25_-P_75_)	65 (59–72)		65 (59–72)		65 (60–72)		−0.27	0.7870
Gender							1.97	0.1601
Male	152	66.7	81	71.1	71	62.3		
Female	76	33.3	33	29.0	43	37.7		
Level of medical Institutions							0.02	0.9895
Tertiary hospital	151	66.2	76	66.7	75	65.8		
Secondary hospital	69	30.3	34	29.8	35	30.7		
Primary hospital	8	3.5	4	3.5	4	3.5		
Region							0.24	0.8873
Eastern region	102	44.7	52	45.6	50	43.9		
Central region	106	46.5	53	46.5	53	46.5		
Western region	20	8.8	9	7.9	11	9.7		
Comorbidity								
High blood pressure	76	33.3	38	33.3	38	33.3	0.00	1.0000
Heart failure	20	8.8	9	7.9	11	9.7	0.22	0.6396
Atrial fibrillation	2	0.9	1	0.9	1	0.9	0.00	1.0000
Heart valve disease	1	0.4	1	0.9	0	0.0	1.00	0.3162
Stroke	14	6.1	6	5.3	8	7.0	0.30	0.5811
Pulmonary embolism	0	0.0	0	0.0	0	0.0	\	
COPD	2	0.9	1	0.9	1	0.9	0.00	1.0000
Chronic nephrosis	2	0.9	2	1.8	0	0.0	2.02	0.1555
Diabetes	74	32.5	38	33.3	36	31.6	0.08	0.7773
Drug combination								
Beta blockers	141	61.8	63	55.3	78	68.4	4.18	0.0409
Nitrate esters	209	91.7	108	94.7	101	88.6	2.81	0.0935
ACEI or ARB	114	50.0	49	43.0	65	57.0	4.49	0.0341
Calcium antagonist	80	35.1	36	31.6	44	38.6	1.23	0.2669
Antiplatelet/thrombolytic/anticoagulant	202	88.6	103	90.4	99	86.8	0.69	0.4046
Lipid regulating agents	200	87.7	99	86.8	101	88.6	0.16	0.6865
Related surgery of CHD	69	30.3	37	32.5	32	28.1	0.52	0.4710

#### Single-Factor Analysis of Outcome Indicators

In the surgery sample, between the salvianolate injection group and Danhong injection group, there were no significant differences in hospitalization duration, 10d rehospitalization rate, 30d rehospitalization rate, 90d rehospitalization rate, and the medical cost per hospitalization (as shown in **Table 3**).

**Table 3:** Comparison of outcomes of salvianolate injection group and Danhong injection group in the surgery sample.

Variable	Total					Salvianolate injection group				Danhong injection group			*Z/χ^2^*	*P*
	(N = 228)					(N = 114)				(N = 114)				
	P_50_		P_25_	P_75_		P_50_	P_25_	P_75_		P_50_	P_25_	P_75_		
Average days of per hospital stay	10		7	12		9	6	11		10	7	13	1.77	0.0766
10d rehospitalization rate, n (%)	3 (1.3)					0 (0.0)				3 (2.6)			3.04	0.0812
30d rehospitalization rate, n (%)	9 (4.0)					4 (3.5)				5 (4.4)			0.12	0.7338
90d rehospitalization rate, n (%)	20 (8.8)					13 (11.4)				7 (6.1)			1.97	0.1601
Medical costs per hospitalization	13170.3	8465.4			42342.5	12900.5	8208.5		42611.6	13578.2	8591.1	42073.4	0.41	0.6784

#### Multi-Factor Analysis of Outcome Indicators

For multiple linear regression models to control potential confounding factors such as age, gender, medical institution level, and comorbidities, in the surgery sample, there was no significant difference between two groups in terms of hospitalization duration.

### Surgery Sample: Salvianolate Injection and Alprostadil Injection

#### Baseline Characterization

After stratification analysis and PSM, 90 pairs of matched pairs were obtained. Among the surgery sample, 90 patients were in the salvianolate injection group and alprostadil injection group. There was a statistically significant difference in the distribution of nitrate esters in the two groups. At the same time, there was no statistically significant difference in the distribution of age, gender, medical institution level, region, comorbidities, other drug combination, and whether or not CHD-related surgery was performed (as shown in **Table 4**).

**Table 4:** Baseline characteristics of the salvianolate injection group and the alprostadil injection group in the surgery sample.

Variable	Total		Salvianolate injection group		Alprostadil injection group		*Z/χ^2^*	*P*
	(N = 180)		(N = 90)		(N = 90)			
	N	%	N	%	N	%		
Age, P_50_ (P_25_-P_75_)	66 (59–72)		64 (59–72)		68 (59–73)		0.59	0.5535
Gender							0.37	0.5428
Male	108	60.0	52	57.8	56	62.2		
Female	72	40.0	38	42.2	34	37.8		
Level of medical institutions							0.03	0.9852
Tertiary hospital	133	73.9	66	73.3	67	74.4		
Secondary hospital	45	25.0	23	25.6	22	24.4		
Primary hospital	2	1.1	1	1.1	1	1.1		
Region							2.67	0.2636
Eastern region	108	60.0	54	60.0	54	60.0		
Central region	54	30.0	30	33.3	24	26.7		
Western region	18	10.0	6	6.7	12	13.3		
Comorbidity							0.02	0.8793
High blood pressure	73	40.6	36	40.0	37	41.1		
Heart failure	21	11.7	10	11.1	11	12.2	0.05	0.8164
Atrial fibrillation	2	1.1	1	1.1	1	1.1	0.00	1.0000
Heart valve disease	1	0.6	0	0.0	1	1.1	1.01	0.3160
Stroke	16	8.9	8	8.9	8	8.9	0.00	1.0000
Pulmonary embolism	0	0.0	0	0.0	0	0.0	\	
COPD	0	0.0	0	0.0	0	0.0	\	
Chronic nephrosis	5	2.8	2	2.2	3	3.3	0.21	0.6501
Diabetes	72	40.0	36	40.0	36	40.0	0.00	1.0000
Drug combination								
Beta blockers	112	62.2	53	58.9	59	65.6	0.85	0.3563
Nitrate esters	174	96.7	84	93.3	90	100.0	6.21	0.0127
ACEI or ARB	96	53.3	45	50.0	51	56.7	0.80	0.3700
Calcium antagonist	64	35.6	27	30.0	37	41.1	2.42	0.1194
Antiplatelet/thrombolytic/anticoagulant	166	92.2	81	90.0	85	94.4	1.24	0.2656
Lipid regulating agents	160	88.9	82	91.1	78	86.7	0.90	0.3428
Related surgery of CHD	41	22.8	23	25.6	18	20.0	0.79	0.3742

#### Single-Factor Analysis of Outcome Indicators

In the surgery sample, average days per hospitalization in the salvianolate injection group was slightly lower than that in the alprostadil group, and the difference was statistically significant (*P* < 0.05). There was no significant difference in the 10d rehospitalization rate, 30d rehospitalization rate, 90d rehospitalization rate, and medical cost per hospitalization between the two groups (as shown in **Table 5**).

**Table 5:** Comparison of outcomes between the salvianolate injection group and the alprostadil injection group in the surgery sample.

Variable	Total			Salvianolate injection group			Alprostadil injection group			*Z/χ^2^*	*P*
	(N = 180)			(N = 90)			(N = 90)				
	P_50_	P_25_	P_75_	P_50_	P_25_	P_75_	P_50_	P_25_	P_75_		
Average days of per hospital stay	9	7	13	9	6	11	10	7	14	2.07	0.0389
10d rehospitalization rate, n (%)	0 (0.0)			0 (0.0)			0 (0.0)			\	
30d rehospitalization rate, n (%)	5 (2.8)			3 (3.3)			2 (2.2)			0.21	0.6501
90d rehospitalization rate, n (%)	19 (10.6)			10 (11.1)			9 (10.0)			0.06	0.8083
Medical cost per hospitalization	13542.4	8570.3	39148.7	12348.1	8047.8	45162.2	14167.3	10071.7	35914.0	0.89	0.3744

#### Multi-Factor Analysis of Outcome Indicators

Multiple linear regression models were applied to control potential confounding factors such as age, gender, medical institution level, and comorbidities. In the surgery sample, the average hospitalization days in the salvianolate injection group was 0.805 times as long as that in the alprostadil injection group, which means less than that in the alprostadil injection group, and the difference was statistically significant (*P* = 0.023).

### Non-Surgery Sample: Salvianolate Injection and Danhong Injection

#### Baseline Characterization

After stratification analysis and PSM, 485 pairs of matched pairs were obtained. Among the surgery sample, 485 patients were in the salvianolate injection group and Danhong injection group. There was a statistically significant difference in the distribution of whether comorbid with pulmonary embolism in the two groups. At the same time, there was no statistically significant difference in the distribution of age, gender, medical institution level, region, other comorbidities, drug combination (as shown in **Table 6**).

**Table 6:** Baseline characteristics of the salvianolate injection group and Danhong injection group in the non-surgery sample.

Variable	Total		Salvianolate injection group		Danhong injection group		*Z/χ^2^*	*P*
	(N = 970)		(N = 485)		(N = 485)			
	N	%	N	%	N	%		
Age, P_50_ (P_25_-P_75_)	67 (59–76)		68 (60–76)		67 (59–76)		−0.96	0.3393
Gender							0.50	0.4783
Male	443	45.7	216	44.5	227	46.8		
Female	527	54.3	269	55.5	258	53.2		
Level of medical institutions							0.04	0.9800
Tertiary hospital	435	44.9	219	45.2	216	44.5		
Secondary hospital	440	45.4	219	45.2	221	45.6		
Primary hospital	95	9.8	47	9.7	48	9.9		
Region							2.47	0.2903
Eastern region	374	38.6	176	36.3	198	40.8		
Central region	516	53.2	265	54.6	251	51.8		
Western region	80	8.3	44	9.1	36	7.4		
Comorbidity								
High blood pressure	224	23.1	120	24.7	104	21.4	1.49	0.2228
Heart failure	56	5.8	33	6.8	23	4.7	1.90	0.1686
Atrial fibrillation	22	2.3	9	1.9	13	2.7	0.74	0.3883
Heart valve disease	1	0.1	0	0.0	1	0.2	1.00	0.3171
Stroke	50	5.2	29	6.0	21	4.3	1.35	0.2454
Pulmonary embolism	4	0.4	0	0.0	4	0.8	4.02	0.0451
COPD	7	0.7	2	0.4	5	1.0	1.30	0.2551
Chronic nephrosis	12	1.2	7	1.4	5	1.0	0.34	0.5613
Diabetes	200	20.6	100	20.6	100	20.6	0.00	1.0000
Drug combination								
Beta blockers	437	45.1	215	44.3	222	45.8	0.20	0.6515
Nitrate esters	696	71.8	336	69.3	360	74.2	2.93	0.0870
ACEI or ARB	434	44.7	219	45.2	215	44.3	0.07	0.7962
Calcium antagonist	347	35.8	185	38.1	162	33.4	2.37	0.1234
Antiplatelet/thrombolytic/anticoagulant	473	48.8	229	47.2	244	50.3	0.93	0.3353
Lipid regulating agents	725	74.7	373	76.9	352	72.6	2.41	0.1207

#### Single-Factor Analysis of Outcome Indicators

In the non-surgical group, the average hospitalization duration in the salvianolate injection group was 9.2 ± 4.5 d, lower than that in the Danhong injection group (9.8 ± 4.8 d), and the difference was statistically significant (*P* < 0.05) (as shown in **Table 7**).

**Table 7:** Comparison of the outcomes of the salvianolate injection group and the Danhong injection group in the non-surgery sample.

Variable	Total			Salvianolate injection group			Danhong injection group			*Z/χ^2^*	*P*
	(N = 970)			(N = 485)			(N = 485)				
	P_50_	P_25_	P_75_	P_50_	P_25_	P_75_	P_50_	P_25_	P_75_		
Average days of per hospital stay	9	7	12	9	7	11	9	7	12	2.12	0.0340
10d rehospitalization rate, n (%)	10 (1.0)			6 (1.2)			4 (0.8)			0.40	0.5249
30d rehospitalization rate, n (%)	22 (2.3)			13 (2.7)			9 (1.9)			0.74	0.3883
90d rehospitalization rate, n (%)	74 (7.6)			32 (6.6)			42 (8.7)			1.46	0.2265
Medical costs per hospitalization	6113.4	4514.9	9104.3	6056.7	4805.2	9038.0	6160.8	4119.0	9184.8	−1.75	0.0794

#### Multi-Factor Analysis of Outcome Indicators

Multiple linear regression models were used to control potential confounding factors such as age, gender, medical institution level, and comorbidities. There was no statistical difference (*P* = 0.199) in the days of hospitalization between the salvianolate group and the Danhong injection group in the non-surgery sample.

### Non-Surgery Sample: Salvianolate Injection and Alprostadil Injection

#### Baseline Characterization

After stratification analysis and PSM, 169 pairs of matched pairs were obtained. Among the surgery sample, 169 patients were in the salvianolate injection group and alprostadil injection group. There was a statistically significant difference in the distribution of region, whether combined drugs with nitrate esters or antiplatelet/thrombolytic/anticoagulation, ACEI or ARB or calcium channel blockers between the two groups. At the same time, there was no statistically significant difference in the distribution of age, gender, medical institution level, region, comorbidities, other combined drugs that did not mentioned above (as shown in **Table 8**).

**Table 8:** Baseline characteristics of salvianolate injection group and alprostadil injection group in the non-surgery sample.

Variable	Total		Salvianolate injection group		Alprostadil injection group		*Z/χ^2^*	*P*
	(N = 338)		(N = 169)		(N = 169)			
	N	%	N	%	N	%		
Age, P_50_ (P_25_-P_75_)	69 (62–78)		70 (61–79)		69 (63–78)		−0.38	0.7045
Gender							2.68	0.1019
Male	157	46.5	71	42.0	86	50.9		
Female	181	53.6	98	58.0	83	49.1		
Level of medical institutions							3.18	0.2043
Tertiary hospital	212	62.7	113	66.9	99	58.6		
Secondary hospital	104	30.8	48	28.4	56	33.1		
Primary hospital	22	6.5	8	4.7	14	8.3		
Region							9.68	0.0079
Eastern region	204	60.4	104	61.5	100	59.2		
Central region	115	34.0	62	36.7	53	31.4		
Western region	19	5.6	3	1.8	16	9.5		
Comorbidity								
High blood pressure	113	33.4	51	30.2	62	36.7	1.61	0.2047
Heart failure	31	9.2	19	11.2	12	7.1	1.74	0.1871
Atrial fibrillation	12	3.6	5	3.0	7	4.1	0.35	0.5566
Heart valve disease	1	0.3	0	0.0	1	0.6	1.00	0.3166
Stroke	18	5.3	8	4.7	10	5.9	0.23	0.6280
Pulmonary embolism		0.0		0.0		0.0		
COPD	3	0.9	0	0.0	3	1.8	3.03	0.0819
Chronic nephrosis	3	0.9	1	0.6	2	1.2	0.34	0.5620
Diabetes	100	29.6	43	25.4	57	33.7	2.78	0.0952
Drug combination								
Beta blockers	178	52.7	87	51.5	91	53.9	0.19	0.6630
Nitrate esters	282	83.4	113	66.9	169	100.0	67.12	<0.0001
ACEI or ARB	178	52.7	80	47.3	98	58.0	3.85	0.0499
Calcium antagonist	154	45.6	68	40.2	86	50.9	3.86	0.0493
Antiplatelet/thrombolytic/anticoagulant	210	62.1	91	53.9	119	70.4	9.86	0.0017
Lipid regulating agents	269	79.6	136	80.5	133	78.7	0.16	0.6856

#### Single-Factor Analysis of Outcome Indicators

In the non-surgery sample, medical cost per hospitalization in the salvianolate injection group was lower than that in the alprostadil group. Moreover, the days of hospitalization at a time in the salvianolate injection group was slightly lower than that in the alprostadil group, and the difference was statistically significant (*P* < 0.05) (as shown in **Table 9**).

**Table 9:** Comparison of outcomes between the salvianolate injection group and the alprostadil injection group in the non-surgery sample.

Variable	Total				Salvianolate injection group			Alprostadil injection group			*Z/χ^2^*	*P*
	(N = 338)				(N = 169)			(N = 169)				
	P_50_		P_25_	P_75_	P_50_	P_25_	P_75_	P_50_	P_25_	P_75_		
Average days of per hospital stay	10		7	14	9	7	12	10	8	14	2.48	0.0130
10d rehospitalization rate, n (%)	5 (1.5)				4 (2.4)			1 (0.6)			1.83	0.1765
30d rehospitalization rate, n (%)	9 (2.7)				6 (3.6)			3 (1.8)			1.03	0.3108
90d rehospitalization rate, n (%)	27 (8.0)				13 (7.7)			14 (8.3)			0.04	0.8410
Medical costs per hospitalization	8472.3		5629.2	11734.7	7718.7	5425.5	10616.4	9462.4	6037.9	12762.7	2.84	0.0045

#### Multi-Factor Analysis of Outcome Indicators

Multiple linear regression models were used to control potential confounding factors such as age, gender, medical institution level, and comorbidities. In the non-surgery sample, the days of per hospitalization of salvianolate injection group were 0.827 times that of the alprostadil group, which was lower than that of the alprostadil group, and the difference was statistically significant (*P* = 0.011). And in the non-surgery sample, the average hospitalization cost of the salvianolate injection group was 0.810 times that of the alprostadil group, which was lower than the alprostadil group, and the difference was statistically significant.

## Discussion

This study focused on the clinical and economic evaluation of salvianolate injection, comparing with alprostadil injection and Danhong injection, which are commonly used in the treatment of CHD (Li and Tang, 2006; Li and Wang, 2009). In this study, we compared the two drugs with salvianolate injection through clinical and economic evaluation outcome indicators such as medical cost of hospitalization, days of hospital stay, and rehospitalization rate (Miao et al., 2006), to evaluate the drug economy and effectiveness of salvianolate injection in the real world. Because the data were not from controlled clinical trials, but from the real-world data, it can provide evidence for the effectiveness and economic effects (Yu and Yan, 2012). As real-world data can provide instructive information for clinical practice (Makady et al., 2017), the results of this study can provide a certain reference for the clinical practice of CHD.

However, real-world data also has certain defects and is prone to selection bias (Dimick and Livingston, 2010). Therefore, stratified analysis and PSM were used to reduce the impact of confounding factors on intervention effect estimation. The baseline situation between the study groups was more balanced and comparable than before (Pourhoseingholi et al., 2012). After PSM, stratified analysis, and multi-factor analysis, this study generated some significant findings that are worth further discussion as below.

Regarding hospitalization duration, it found that the hospitalization duration of salvianolate injection group was significantly shorter than that of Danhong injection group in the non-surgery sample. The hospitalization duration of salvianolate injection group was significantly shorter than those of alprostadil injection group in both surgery and non-surgery samples. Past research found that salvianolate injection was most frequently combined with clopidogrel and isosorbide dinitrate, and such combination showed higher clinical effectiveness as compared with other drug combinations (Chang et al., 2013). In particular, compared with conventional treatment for the treatment of CHD, the combination of salvianolate injection and conventional treatment was associated with a reduction in hospital stay (Dong et al., 2018). All of these findings show that clinical application of salvianolate injection has comparative advantages in reducing hospitalization stay of CHD patients.

Regarding medical cost per hospitalization, this study just found that the medical cost per hospitalization of salvianolate injection group was significantly (*P* < 0.05) lower than that of alprostadil injection group in the non-surgery sample. As for CHD patients, surgery often accounts for most of the medical cost of hospitalization. Therefore, application of salvianolate injection can only show its economic advantages for CHD inpatients in non-surgery group when compared with alprostadil injection.

For rehospitalization rates, it found no significantly statistical differences in salvianolate injection group vs. alprostadil injection group or salvianolate injection group vs. Danhong injection group in both surgery and non-surgery samples. It has been indicated that the risk factors for rehabilitation of CHD patients can be much related to body composition (Chen et al., 2017). Such kind of results implies that drug therapies for CHD treatment needs more patient-centered exploration.

This study has several strengths. Firstly, this study was based on real-world data. By using real-world data, the clinical and economic evaluation of salvianolate injection for CHD was conducted, which can better show the actual clinical practice. Secondly, the data of this study was collected from the National Urban Basic Medical Insurance Database, which covered the whole nation, so the findings have more generalizing significance for CHD treatment. Thirdly, Peng et al. mentioned in their previous study of cost-consequence analysis of salvianolate injection for the treatment of CHD that their study did not stratify the severity of CHD due to the limitation of the original medical record (Dong et al., 2018). Therefore, this study considered the severity of the patient’s disease on the results. Subgroups were carried out to analyze the injection of salvianolate and the other two contrast drugs in the surgery sample and the non-surgery sample, respectively, to make the results more authentic and reliable.

However, at the same time, there were some limitations, which are worth improving in future research. Firstly, since this study was a retrospective study, and the baseline variables provided were limited, potential confounders were difficult to control. Although stratified analysis and PSM were used, the interference of confounders and the selective bias cannot be eliminated entirely. Secondly, although the data of this study came from the National Urban Basic Medical Insurance Database, the sample size that met the rigor enrollment and medication conditions was relatively small, which led to the failure of multi-factor analysis of part of the rehospitalization rate. Thirdly, the data source in this study is unable to provide sufficient economic and effectiveness indicators due to its data limitations, which may lead to some bias to some extent. The prospective design of cost-effectiveness analysis is suggested for future evaluation of salvianolate injection.

## Conclusion

Salvianolate injection showed advantages in reducing hospitalization duration for inpatients with CHD when comparing with alprostadil injection and Danhong injection. The results of this real-world study can help to inform prescription practice for CHD patients. More prospective investigation can be employed in future studies on the effectiveness of salvianolate injection.

## Data Availability Statement

The raw data supporting the conclusions of this article will be made available by the authors, without undue reservation, to any qualified researcher.

## Ethics Statement

The studies involving human participants were reviewed and approved by: The research was reviewed and approved by the Ethics Committee of Peking University (71603008). Written informed consent for participation was not required for this study in accordance with the national legislation and the institutional requirements.

## Author Contributions

HH and SH designed the study. XC and HZ performed the statistical analysis. LY and XC wrote the first draft of the manuscript. SH and HZ gave conceptual advice. LY and HH revised the final draft. All authors contributed to the article and approved the submitted version.

## Conflict of Interest

The authors declare that the research was conducted in the absence of any commercial or financial relationships that could be construed as a potential conflict of interest.

The handling editor declared a shared affiliation, though no other collaboration with several of the authors XC, HZ, SH, at the time of the review.
